# Hypoxic preacclimatization combining intermittent hypoxia exposure with physical exercise significantly promotes the tolerance to acute hypoxia

**DOI:** 10.3389/fphys.2024.1367642

**Published:** 2024-04-03

**Authors:** Jiaxin Xie, Shenwei Xie, Zhifeng Zhong, Huaping Dong, Pei Huang, Simin Zhou, Huaijun Tian, Jijian Zhang, Yu Wu, Peng Li

**Affiliations:** ^1^ Department of High Altitude Operational Medicine, College of High Altitude Military Medicine, Army Medical University (Third Military Medical University), Chongqing, China; ^2^ Department of Health Management, The 953rd Hospital of PLA, Shigatse, China

**Keywords:** hypoxia, high altitude, preacclimatization, hypobaric chamber, acute mountain sickness

## Abstract

**Background:** Both hypoxia exposure and physical exercise before ascending have been proved to promote high altitude acclimatization, whether the combination of these two methods can bring about a better effect remains uncertain. Therefore, we designed this study to evaluate the effect of hypoxic preacclimatization combining intermittent hypoxia exposure (IHE) and physical exercise on the tolerance to acute hypoxia and screen the optimal preacclimatization scheme among the lowlanders.

**Methods:** A total of 120 Han Chinese young men were enrolled and randomly assigned into four groups, including the control group and three experimental groups with hypoxic preacclimatization of 5-day rest, 5-day exercise, and 3-day exercise in a hypobaric chamber, respectively. Main physical parameters for hypoxia acclimatization, AMS incidence, physical and mental capacity were measured for each participant in the hypobaric chamber simulated to the altitude of 4500 m in the effect evaluation stage. The effect was compared between different schemes.

**Results:** During the effect evaluation stage, SpO_2_ of the 5-day rest group and 5-day exercise group was significantly higher than that of the control group (*p* = 0.001 and *p* = 0.006, respectively). The participants with 5-day rest had significantly lower HR than the controls (*p* = 0.018). No significant differences of AMS incidence were found among the four groups, while the proportion of AMS headache symptom (moderate and severe vs. mild) was significantly lower in the 3-day exercise group than that in the control group (*p* = 0.002). The 5-day exercise group had significantly higher VO_2_max, than the other three groups (*p* = 0.033, *p* < 0.001, and *p* = 0.023, respectively). The 5-day exercise group also had significantly higher digital symbol and pursuit aiming test scores, while shorter color selection reaction time than the control group (*p* = 0.005, *p* = 0.005, and *p* = 0.004, respectively).

**Conclusion:** Hypoxic preacclimatization combining IHE with physical exercise appears to be efficient in promoting the tolerance to acute hypoxia. Hypoxia duration and physical exercise of moderate intensity are helpful for improvement of SpO_2_ and HR, relief of AMS headache symptoms, and enhancement of mental and physical operation capacity.

## Introduction

Plateau tourism is becoming increasingly popular worldwide, with Tibet alone attracting over 24 million domestic and foreign visitors from January to June 2023. In addition to tourism, many individuals engage in work, business, or sports activities at high altitudes, such as skiing, trekking, and climbing. All these people have to deal with the challenges of high altitude hypoxia which induces various mountain sicknesses and impairs the mental and physical capacity of the natives and immigrants, especially among those with acute hypoxia exposure (Netzer et al., 2013; Faulhaber et al., 2021; Forrer et al., 2021). Pharmacological prophylaxis by acetazolamide and modern preacclimatization strategies may be effective preparation options for the lowlanders to high altitude (Burtscher et al., 2023). The use of acetazolamide has been proved to be effective on preventing acute mountain sickness (AMS), while it needs the travel medicine practitioners’ professional judgment on the reasonable medication time as well as dosage to avoid adverse drug reactions (Ritchie et al., 2012; Shlim, 2020). Hypoxic preacclimatization through continuous or intermittent hypoxia exposure (CHE or IHE) is increasingly applied to enhance acclimatization to high altitude.

CHE is usually to live in a chamber simulating different high altitudes for a few days and thereafter achieve a certain degree of hypoxic acclimatization, but participants may experience AMS akin to the real ascending to high altitudes. Furthermore, this approach is time-consuming and unsuitable for emergency procedures conducted at high altitudes. IHE refers to periodic short-term and repeated exposure to hypoxic environment equivalent to a certain altitude and the acquirement of resistance to subsequent longer or more serious hypoxia injury (Viscor et al., 2018). The training method known as ‘living high, training low’ involves athletes residing in real high altitude or artificial hypoxic environment and then exercising at lower elevations. This approach has been gaining attention from the global sports community due to its positive impact on athletic performance (Inness et al., 2017; Yang et al., 2018). Currently, hypoxic preacclimatization with other IHE patterns, i.e., breathing in a hypobaric or normobaric hypoxic room, or inhaling low oxygen mixtures through masks has also been widely conducted. Although the exact mechanisms still remain unclear, it is believed that IHE can trigger compensatory physical changes primarily in oxygen metabolism pattern and the subsequent improvement of oxygen-carrying capacity (Nagel et al., 2020; Wojan et al., 2023). IHE with hypobaric chamber has been mainly used for animal researches, in which increased circulating erythropoietin levels and hypoxic ventilatory response (HVR) was observed (Logan et al., 2016; Coimbra-Costa et al., 2021). It could also trigger antioxidant defense and impact intracellular signal transduction as well as inflammatory immune response (Aguilar et al., 2018; Yan et al., 2021).

Impairment of mental and physical capacity caused by hypoxia exposure has been widely reported, and hypoxic preacclimatization may be an effective measure to mitigate such damage. Acute IHE in hypobaric chambers has shown obvious protective effects on cardiovascular system, such as the effects against hypoxic hypertension and myocardial ischemia-reperfusion injury (Cui et al., 2012; Terada et al., 2021). Chronic IHE treatment adaptively regulates cardiac autonomic activity (Shi et al., 2020) and increases antioxidant capacity in the brain (Costa et al., 2013). A recent meta-analysis concluded that exercising under hypoxic environment could significantly improve various cognitive functions, and the effect may be moderated by individual characteristics and/or hypoxic preconditioning parameters (Jung et al., 2020). Our recent study also suggests that the remote ischemic preconditioning (RIPC) treatment can notably enhance spatial memory and sleep quality in subjects exposed to acute hypoxia, potentially resulting in improved performance at high altitude (Wu et al., 2023). The effect of hypoxic preacclimatization with hypobaric chamber on cognitive function remains to be evaluated. Additionally, prior studies on IHE have recommended different schemes regarding the essential elements such as the degree of hypoxia, frequency, and duration of hypoxia exposure, and also the application of hypobaric or normobaric hypoxia (Koehle et al., 2008; Navarrete-Opazo et al., 2017), while no universally recognized standards have been established in this regard.

The protective effect of hypoxic preacclimatization with hypobaric chamber against hypoxia remains inconclusive, particularly in relation to performance in hypoxic environment. Therefore we designed this study to assess the effect of hypoxic preacclimatization combining IHE and physical exercise with hypobaric chamber on the subsequent tolerance to acute hypoxia exposure and screen the optimal schemes among the lowlanders.

## Materials and methods

### Study design and participants

**FIGURE 1:**
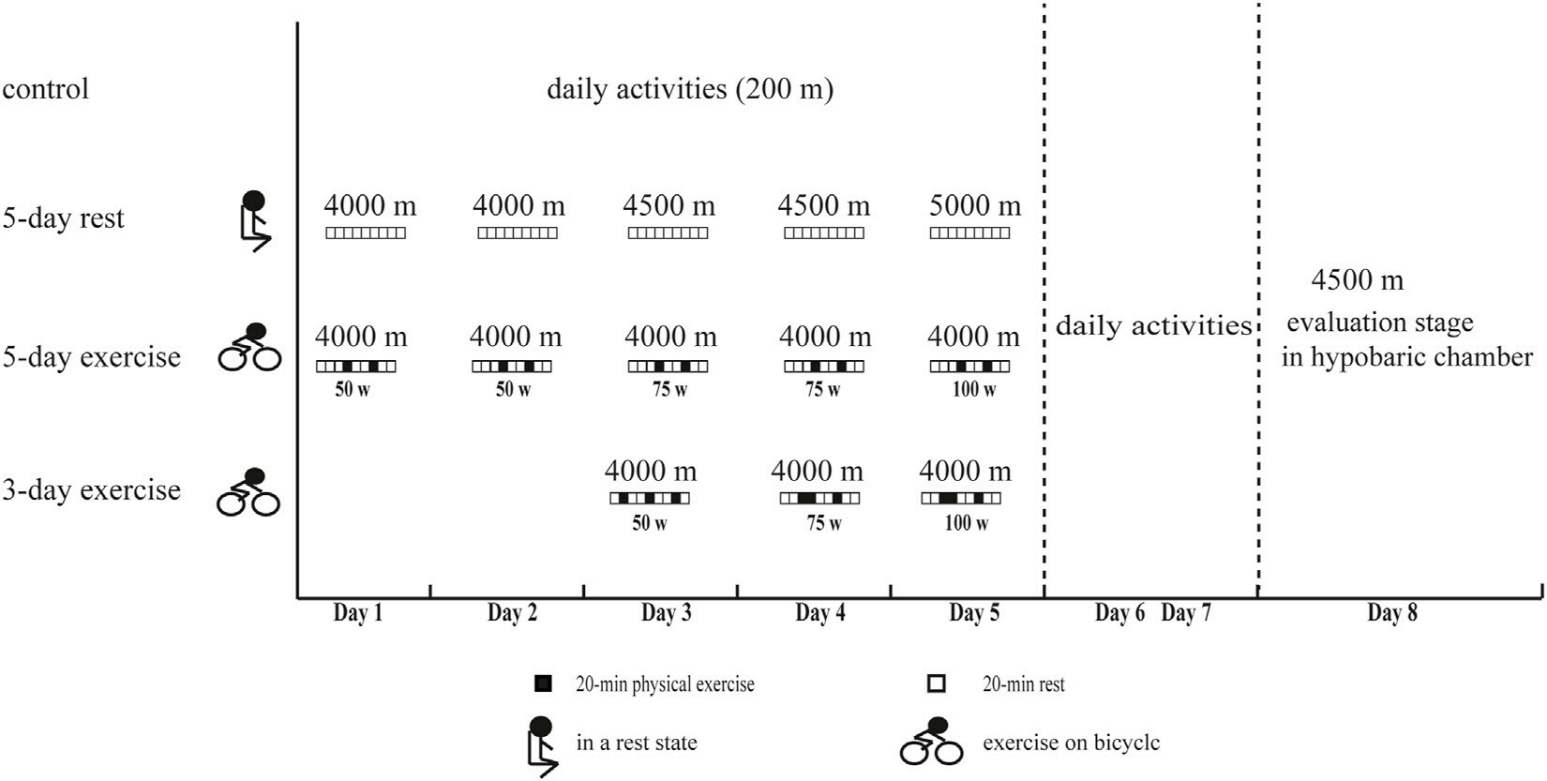
The experimental procedure of this study. 5-day rest: including IHE consisting of 3-h hypoxic exposure for five consecutive days, simulated altitude of 4000 m on the first and second days, 4500 m on the third and fourth days, and 5000 m on the fifth day, and rest state. 5-day exercise: including IHE consisting of 3-h hypoxic exposure for five consecutive days, simulated altitude of 4000m, and state of physical exercise with 50W on the first to second day, 75W on the third to fourth day, and 100W on the fifth day. The exercise was split into two 20-min sessions separated by a 40-min break. 3-day exercise: including IHE consisting of 4-h hypoxic exposure for three consecutive days, simulated altitude of 4000m, and the state of physical exercise with 50W on the first day, 75W on the second day, and 100W on the third day. The exercise was split into three 20-min sessions, each separated by a 40-min break.

A total of 120 healthy young men aged from 18 to 24 years were recruited from September to November 2020. They were randomly and equally assigned to four groups, including the control group and three experimental groups (5-day rest, 5-day exercise, and 3-day exercise in a hypobaric chamber). All the participants had never been exposed to high altitude. In the first stage, participants of the three experimental groups underwent hypoxic preacclimatization with different schemes in a hypobaric chamber. Participants of the 5-day rest group were exposed to IHE consisting of 3-h hypoxic exposure for five consecutive days. The altitude simulation in the hypobaric chamber increased gradually, reaching 4000 m on the first and second days, 4500 m on the third and fourth days, and 5000 m on the fifth day. They were all in a resting state during this stage. Participants of 5-day exercise group were exposed to IHE consisting of 3-h hypoxic exposure for five consecutive days. The simulated altitude in the hypobaric chamber was 4000 m, and the personnel were in a state of physical exercise during this stage. The exercise load is set using a Cosmed’ magnetic brake bicycle ergometers. The exercise load is 50W on the first to second day, 75W on the third to fourth day, and 100W on the fifth day. The exercise was split into two 20-min sessions separated by a 40-min break. Participants of 3-day exercise group were exposed to IHE consisting of 4-h hypoxic exposure for three consecutive days. The simulated altitude was 4000 m, and the personnel were in a state of physical exercise. The exercise load was set to 50W on the first day, 75W on the second day, and 100W on the third day. The exercise was split into three 20-min sessions, each separated by a 40-min break. The controls stayed in normoxic environment without any forms of hypoxic preacclimatization in this stage. During the effect evaluation stage, participants from the experimental groups re-entered the hypobaric chamber simulating high altitude of 4500 m 2 days after their hypoxic preacclimatization, and the controls received the same evaluation (Figure 1). The hypoxic physiological parameters, AMS incidence, cognitive function, and aerobic exercise capacity were measured and then compared between groups. The final numbers of participants who completed the effect evaluation were 29, 30, 29, and 30 for the control group, experimental group of 5-day rest, 5-day exercise, and 3-day exercise, respectively. The protocol conformed to the 1975 Declaration of Helsinki and was approved by the ethics committee of Army Medical University, Chongqing, China. Written informed consents were obtained from all the participants.

### Physiological parameters measurement

During the effect evaluation stage, two skilled physicians measured and recorded basic physiological parameters, including pulse oxygen saturation (SpO_2_), heart rate (HR), systolic blood pressure (SBP), and diastolic blood pressure (DBP) for all the participants 15 min after the simulated altitude rising to 4500 m in the hypobaric chamber. SpO_2_ and HR were measured using a TuffSat Handheld Pulse Oximeter (GE Healthcare, Chicago, IL, United States). SBP and DBP were measured with sphygmomanometers (HEM-6200, OMRON, Beijing, China).

### Acute mountain sickness diagnosis and gradation

AMS was diagnosed and gradated according to the Chinese AMS Scoring system (CAS, GJB-1098-1991). Symptoms in the questionnaire included headache, vomiting/nausea, palpitation, short breath, vertigo, insomnia, drowsiness, decreased food appetite, abdominal distension, diarrhea, constipation, lip cyanosis, hand and foot numbness. Headache and vomiting/nausea were divided into four grades and assigned 1, 2, 4, and 7 points, respectively, and the other symptoms were recorded as 0 or 1 point each. The questionnaires were distributed to the participants beforehand and filled in according to the above symptoms. AMS was divided into 4° according to the total score: essentially no reaction (total score<5 points), mild reaction [headache (+) or vomiting (+) or total score of 5–10 points], moderate reaction [headache (++) or vomiting (++) or total score between 11 and 15 points], and severe reaction [headache (+++) or vomiting (+++) or total score ≥16]. Mild reaction was considered to be positive for AMS.

### Maximum oxygen uptake measurement

The effect of hypoxic preacclimatization on aerobic exercise capacity was evaluated by maximum oxygen uptake ( $\dot{V}$ O_2_max). In this study, $\dot{V}$ O_2_max was predicted by measuring submaximal VO_2_ with Cosmed Fitmate (FM). We designed an incremental exercise test with Cosmed’ magnetic brake bicycle ergometers and instructed the participants to exercise on bicycles in the hypobaric chamber. The intensity was initially set to 0 W, and then gradually increased by 30 W per minute. The cycling frequency was maintained between 50 and 60 revolutions per minute (rpm) throughout the exercise. Oxygen uptake was continuously measured and HR was continuously recorded. Exercising was terminated when there was no further increase in oxygen uptake despite a further increase in intensity or when the heart rate was age-predicted HRmax (85%*(220 - age in years)). Then the value of $\dot{V}$ O_2_max was predicted according to the instrument’s own algorithm. In the whole process of exercise, we continuously recorded HR for each participant using an HR monitor (Polar H10, Polar Electro Inc., Lake Success, NY, United States of America).

### Cognitive functions tests

Each participant received six neurobehavioral tests, including *Benton Visual Retention test*, *Digit Symbol test*, *Pursuit Aiming test*, *Color Selective Reaction test*, *Audible Simple Reaction test*, and *Spatial Memory test*. The former three tests were from the World Health Organization (WHO)-recommended Neurobehavioral Core Test Battery (NCTB), and were used to evaluate visual memory, perceptual speed, and motor skills, respectively (Shanjun et al., 2020). The other three tests were used for testing color selective reaction times, audible simple reaction times, and spatial memory ability. The instruments and methods were the same as described in our previous study (Zhong et al., 2021). The cognitive functions in hypoxic environment were mainly assessed by these tests results and had been demonstrated to be vulnerable at high altitude.

### Statistical analysis

Data in this study was processed and analyzed using the Statistical Program for Social Sciences (SPSS22.0 for Windows, SPSS, Chicago, IL). Differences of categorical variables, such as AMS and headache incidence between groups were evaluated by Chi-square test. Quantitative variables of normal distribution, such as age, SpO_2,_ HR, and BP were shown as mean ± standard deviation, and the difference among groups was compared with one-way ANOVA analysis. Non-normally distributed variables, such as cognitive function tests results and AMS symptoms scores were presented as median (P25, P75). Kruskal–Wallis rank sum test was used to compare the average levels among the multiple groups, and the pairwise comparisons were conducted by Mann-Whitney U test. Comparisons between multiple groups were performed and Bonferroni correction was applied. All statistical tests were two-sided and *p* values less than 0.05 were considered as statistically significant.

## Results

### Baseline characteristics

A total of 118 participants completed the effect evaluation, including 29, 30, 29, and 30 in the control group and experimental groups with 5-day rest, 5-day exercise, and 3-day exercise, respectively. They were all male and the average age (years) for the four groups was 20.67 ± 1.90, 19.87 ± 1.53, 19.73 ± 1.39, and 20.10 ± 1.69, respectively (*p* = 0.133). The body mass index (BMI, kg/m^2^) and education background were also comparable among the four groups (*p* = 0.394 and *p* = 0.741, respectively).

### Effect of hypoxic preacclimatization on physiological parameters

**FIGURE 2:**
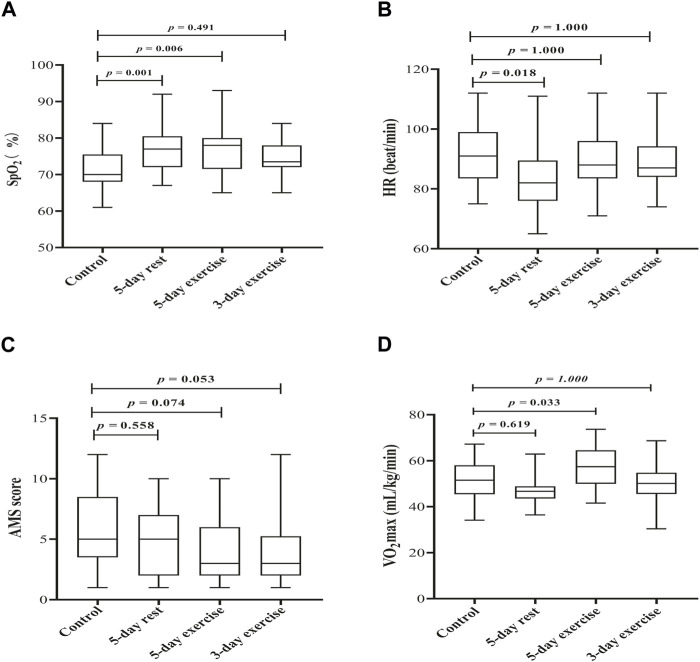
The effect of hypoxic preacclimatization of different schemes on **(A)** SpO_2_ **(B)** HR, **(C)** AMS score, and **(D)** VO_2_max, respectively. SpO_2_: pulse oxygen saturation, HR: heart rate, AMS: acute mountain sickness, VO_2_max: maximum oxygen uptake.

The average SpO_2_ (%) of the control group and experimental groups with 5-day rest, 5-day exercise, and 3-day exercise was 71.55 ± 5.45, 77.34 ± 6.14, 76.55 ± 6.37, and 74.13 ± 4.46, respectively. Compared with the control group, the experimental groups with 5-day rest and 5-day exercise had significantly higher SpO_2_ levels (*p =* 0.001 and *p =* 0.006, respectively). However, the difference of SpO_2_ between the control group and experimental group with 3-day exercise in the hypobaric chamber did not reach the significant level (*p* = 0.491) (Figure 2A).

The average HRs (beat/min) for the control group and experimental groups with 5-day rest, 5-day exercise, and 3-day exercise were 90.93 ± 10.24, 83.31 ± 10.77, 89.44 ± 8.72, and 88.63 ± 8.43, respectively. Participants who had been trained with 5-day rest in the hypobaric chamber had the lowest HR, which was significantly lower than that of the control group (*p* = 0.018). The mean HRs between the other two experiment groups and the control group were not significantly different (*p* = 1.000 for each) (Figure 2B).

### Effect of hypoxic preacclimatization on AMS

The incidence of AMS was not significantly different among the four groups (*p* = 0.198). No significant difference of AMS headache symptom incidence was found among the four groups (*p* = 0.497). After dividing the headache symptom into four grades, however, it was found that the proportions of moderate and severe headache (*versus*. mild headache) in the groups with 5-day exercise and 3-day exercise were significantly lower than that in the control group (*p* = 0.016 and *p* = 0.002, respectively) (Table 1).

**TABLE 1:** The incidence of AMS and headache symptom among the four groups during the effect evaluation stage [n (%)].

AMS and headache symptom		Controls	Participants with hypoxic preacclimatization			χ^2^	*p*-Value
			5-day rest	5-day exercise	3-day exercise		
AMS (CAS)	No	12 (41.38)	14 (46.67)	17 (58.62)	20 (66.67)		
	Mild	15 (51.72)	16 (53.33)	12 (41.38)	9 (30.00)	4.670^a^	0.198
	Moderate	2 (6.90)	0 (0.00)	0 (0.00)	1 (3.33)		
Headache	No	3 (10.34)	7 (23.33)	6 (20.69)	4 (13.33)	2.382^a^	0.497
	Mild	5 (17.24)	8 (26.67)	12 (41.38)	16 (53.33)		
	Moderate	13 (44.83)	12 (40.00)	8 (27.59)	7 (23.33)	11.088^b^	**0.011**
	Severe	8 (27.59)	3 (10.00)	3 (10.34)	3 (10.00)		

^a^ The difference of AMS, or headache incidence among the four groups.

^b^ The difference of moderate and severe headache (*versus* non-headache) among the four groups. AMS: acute mountain sickness, CAS: Chinese AMS, scoring system.

AMS scores of the experimental groups with 5-day exercise and 3-day exercise in the hypobaric chamber were lower than those of the control group, however, the differences were not significant (*p* = 0.074 and *p* = 0.053, respectively) (Figure 2C).

### Effect of hypoxic preacclimatization on $\dot{V}$ O_2_max

The average $\dot{V}$ O_2_max (mL/kg/min) of the control group, experimental groups with 5-day rest, 5-day exercise, and 3-day exercise in the hypobaric chamber was 51.02 ± 8.38, 47.03 ± 6.52, 57.33 ± 8.77, and 50.49 ± 8.35, respectively. As shown in Figure 2D, participants with 5-day exercise had the highest level of $\dot{V}$ O_2_max when exposed to acute hypoxia, which was significantly higher than that of the control group, experimental groups of 5-day rest and 3-day exercise (*p =* 0.033, *p =* 0.000, and *p =* 0.023, respectively). $\dot{V}$ O_2_max of the groups with 5-day rest and 3-day exercise was not significantly different from that of the control group (*p* = 0.619 and *p* = 1.000, respectively).

### Effect of hypoxic preacclimatization on cognitive functions

The average scores of digital symbol test, pursuit aiming test, and color selective reaction test were significantly different among the four groups (*p* = 0.004, *p =* 0.006*, p* = 0.015, respectively) (Table 2). Pairwise comparisons showed that participants of experimental group with 5-day exercise in the hypobaric chamber had higher average scores of digital symbol test and pursuit aiming test, while shorter color selection reaction time than the controls did (*p* = 0.005, *p* = 0.005, and *p* = 0.004, respectively). The differences of average scores of digital symbol test and color selection reaction time were also significant between the experimental group with 5-day exercise and 5-day rest after correction (*p* = 0.004 and *p* = 0.007, respectively). Compared with the control group and experimental group with 5-day rest, the average scores of digital symbol test for the experimental group with 3-day exercise were higher without significant differences (*p* = 0.174 and *p* = 0.204).

**TABLE 2:** The cognitive performance in hypoxic environment simulated to high altitude of 4500 m [Median (P25, P75)].

Cognitive function tests	Controls	Participants with hypoxic preacclimatization in the hypobaric chamber			χ^2^	*p*-Value
		5-day rest	5-day exercise	3-day exercise		
Benton visual retention	10 (9, 10)	10 (10, 10)	10 (10, 10)	10 (9.5, 10)	7.003	0.072
Digital symbol	51.50 (37.25, 68.00)	54.00 (35.75, 71.00)	68.50 (57.25, 85.00)^*,#^	65.00 (55.00, 76.00)	13.302	**0.004**
Pursuit aiming	112.25 (102.20, 120.75)	120.25 (105.75, 130.63)	135.00 (114.63, 142.38)^*^	119.00 (106.50, 134.75)	12.358	**0.006**
Color selective reaction	0.64 (0.58, 0.76)	0.65 (0.59, 0.69)	0.58 (0.54, 0.65)^*,#^	0.65 (0.57, 0.76)	10.464	**0.015**
Audible simple reaction	0.19 (0.18, 0.23)	0.19 (0.17, 0.20)	0.19 (0.18, 0.21)	0.20 (0.18, 0.22)	2.867	0.413
Lifting agility test with habitual hand	18.50 (15.25, 20.00)	19.00 (15.00, 20.00)	19.00 (17.00, 21.50)	17.00 (15.50, 20.00)	2.553	0.466
Spatial memory	10.50 (7.25, 12.00)	10.00 (8.00, 11.00)	9.00 (8.00, 11.00)	9.00 (7.50, 11.50)	1.077	0.783

^*^, ^#^, Significantly different from the controls and the experimental group with 5-day rest after correction, respectively.

Bold values represent significance.

## Discussion

We conducted a population-based study for the first time to evaluate the effect of hypoxic preacclimatization combining IHE with physical exercise in a hypobaric chamber on the subsequent tolerance to acute hypoxia among the lowlanders. IHE leads to an increase of blood oxygen saturation primarily due to effective ventilatory acclimatization (Katayama et al., 2001; Yoon et al., 2021). SpO_2_ level is not only the reflection of pulmonary oxygenation function and oxygen carrying capacity of hemoglobin under hypoxic environment, but also serves as a common noninvasive parameter for monitoring exercise intensity during hypoxic trainings (Woorons and Richalet, 2021). A 7-week IHE under hypobaric pressure with mean total exposure time of 30.8 h has been reported to increase SaO_2_ by 4.1% at 4570 m (Hetzler et al., 2009). We also observed an average increase of over 5 percentage points of SpO_2_ among the groups with 5-day rest and 5-day physical exercise as compared with the control group. Hypoxic preacclimatization has probably resulted in an acclimatization response with shorter time than predicted. Nevertheless, it should be noted that although SpO_2_ is significantly correlated with SaO_2_ in most cases, it may not be reliable surrogate marker for SaO_2_ in critically ill patients as well as in hypoxemia for increased bias and decreased accuracy and precision (Hassan et al., 2017; Kanz et al., 2018). HR of the subjects with 5-day rest was significantly lowered as compared with the controls, however, no matter 5-day or 3-day physical exercise in the hypobaric chamber seemed to have no obvious effect on HR. When exercising at certain intensity, the changes of HR among the subjects with IHE training are smaller than those without hypoxic training, indicating that IHE can compensate for the decrease of HR by increasing cardiac output and improve the cardiovascular function (Fukuda et al., 2010). The findings suggested that longer exposure to hypoxia for preacclimatization was more effective in increasing blood oxygen saturation and decreasing heart rate compared to engaging in more intense physical exercise.

Despite the well-established efficacy of IHE in enhancing high altitude hypoxia tolerance and the majority of findings supporting its positive effect on preventing AMS, the conclusions remain inconsistent. It has been reported that hypoxic preacclimatization for 1 h a day for 7 consecutive days had no effect on preventing AMS when entering an altitude of 4500 m. (Faulhaber et al., 2016). In this study, those with hypoxic preacclimatization of 3-day exercise in the hypobaric chamber had the lowest AMS incidence of 33.33%, while the difference was not significant. The physiological changes of cardiovascular, respiratory, and nervous systems induced in three or 5 days’ hypoxic preacclimatization may be insufficient to compensatory for the drastic decrease in ambient oxygen partial pressure.

IHE not only alleviates neurological symptoms associated with AMS, but also enhances cognitive function and uplifts mood at high altitudes (Heinrich et al., 2019; Behrendt et al., 2022). Exercise can also improve cognitive performance in both hypoxic and normoxic condition, although the beneficial effect under severe hypoxia may be attenuated (Lefferts et al., 2016; Komiyama et al., 2017; Li et al., 2017). The cognitive performance during exercising under acute hypoxia is primarily determined by alterations in cerebral blood flow, cerebral metabolism, and possibly neurotransmitter function (Ando et al., 2020). We found that 5-day IHE with physical exercise showed significant effect on perceptual speed, motor skills, and color selective reaction times. Hypoxia exposure of longer duration probably interacted positively with physical exercise of moderate intensity in improving hypoxic tolerance of the brain tissue. During this process, a variety of kinase cascades in brain cells may be initiated and induce changes in chromatin structure through epigenetic mechanisms (Rybnikova and Samoilov, 2015). The possible mechanisms also include activation of transcription factors, such as HIF and changes in expression of a variety of regulatory proteins (Qiao et al., 2014; Wu et al., 2019).

IHE enhances both $\dot{V}$ O_2_max and lactate tolerance, leading to a significant improvement in the aerobic exercise ability of athletes (Gore et al., 2007). A 9-day hypobaric hypoxia exposure at simulated altitude from 4000 m to 5500 m has significantly activated the erythropoietic response and improved the aerobic capacity (Rodriguez et al., 1999). Hypoxic preacclimatization of 5-day exercise in this study also led to a significant increase in $\dot{V}$ O_2_max. In general, prolonged exposure to hypoxia combined with moderate-intensity physical exercise is more effective in enhancing both mental and physical performance in a hypoxic environment.

The duration of each session and the fraction of inspiration O_2_ (FIO2) have been considered as the most effective regimens of hypoxic preacclimatization, while the optimal schemes are still unclear and require further study (Tannheimer and Lechner, 2020). It has been implicated that the effect of hypoxic preacclimatization schemes with short duration was not different between rest and exercise while the schemes with exercise and longer duration could greatly enhance the exercise performance at high altitude (Rieger et al., 2017). In this study, we found that intermittent hypoxic training consisting of 3-h hypoxic exposure for five consecutive days combined with physical exercise was the most effective in promoting hypoxic acclimatization, especially for improvement of physical and mental capacity. The duration of hypoxia is longer than that reported in other studies (Holliss et al., 2014; Taralov et al., 2018). It implies that long duration of hypoxia and physical exercise can probably improve the effect of hypoxic preacclimatization.

This study also has several limitations. Firstly, we failed to collect the intact data on the related physiological parameters, mental and physical capacity before preacclimatization training, and we could not accurately evaluate the comparability between the control and experimental groups although they were matched in some key characteristics, such as age, sex, and history of high altitude exposure. Secondly, we did not conduct field effect evaluation at high altitude. Thirdly, we used the Chinese AMS scoring system (CAS) instead of the better known Lake Louise Score (LLS) in the present study, because the CAS is more commonly used in China, and we did not consider the use of two scoring systems in our study design. Fourthly, we failed to investigate the duration of effect maintenance, which is actually an important aspect of hypoxic preacclimatization. Lastly, as this study was conducted in Chinese Han people, the application audience was relatively limited.

Generally, hypoxic preacclimatization combining IHE with physical exercise in hypobaric chamber could significantly promote the subsequent hypoxic tolerance among the lowlanders, including improvement of SpO_2_ and HR, relief of headache symptoms, and enhancement of mental and physical operation capacity. The schemes consisted of hypoxia degree simulated to the altitude of ≥4000m, duration of ≥3 days, single exposure for ≥3 h, and the total training time of ≥10 h may induce the best effect. Furthermore, longer duration of IHE combined with physical exercise of moderate intensity would be preferable for those with high altitude operations with certain intensity.

## Data Availability

The original contributions presented in the study are included in the article/Supplementary material, further inquiries can be directed to the corresponding authors.

## References

[B1] Aguilar M. Gonzalez-Candia A. Rodriguez J. Carrasco-Pozo C. Canas D. Garcia-Herrera C. (2018). Mechanisms of cardiovascular protection associated with intermittent hypobaric hypoxia exposure in a rat model: role of oxidative stress. Int. J. Mol. Sci. 19, 366. 10.3390/ijms19020366 29373484 PMC5855588

[B2] Ando S. Komiyama T. Sudo M. Higaki Y. Ishida K. Costello J. T. (2020). The interactive effects of acute exercise and hypoxia on cognitive performance: a narrative review. Scand. J. Med. Sci. Sports 30, 384–398. 10.1111/sms.13573 31605635

[B3] Behrendt T. Bielitzki R. Behrens M. Herold F. Schega L. (2022). Effects of intermittent hypoxia-hyperoxia on performance- and health-related outcomes in humans: a systematic review. Sports Med. Open 8, 70. 10.1186/s40798-022-00450-x 35639211 PMC9156652

[B4] Burtscher J. Swenson E. R. Hackett P. H. Millet G. P. Burtscher M. (2023). Flying to high-altitude destinations: is the risk of acute mountain sickness greater? J. Travel Med. 30, taad011. 10.1093/jtm/taad011 36694981 PMC10289512

[B5] Coimbra-Costa D. Garzon F. Alva N. Pinto T. C. C. Aguado F. Torrella J. R. (2021). Intermittent hypobaric hypoxic preconditioning provides neuroprotection by increasing antioxidant activity, erythropoietin expression and preventing apoptosis and astrogliosis in the brain of adult rats exposed to acute severe hypoxia. Int. J. Mol. Sci. 22, 5272. 10.3390/ijms22105272 34067817 PMC8156215

[B6] Costa D. C. Alva N. Trigueros L. Gamez A. Carbonell T. Rama R. (2013). Intermittent hypobaric hypoxia induces neuroprotection in kainate-induced oxidative stress in rats. J. Mol. Neurosci. 50, 402–410. 10.1007/s12031-012-9945-8 23288703

[B7] Cui F. Gao L. Yuan F. Dong Z. F. Zhou Z. N. Kline D. D. (2012). Hypobaric intermittent hypoxia attenuates hypoxia-induced depressor response. PLoS One 7, e41656. 10.1371/journal.pone.0041656 22848558 PMC3407201

[B8] Faulhaber M. Grobner K. Rausch L. Gatterer H. Menz V. (2021). Effects of acute hypoxia on lactate thresholds and high-intensity endurance performance-A pilot study. Int. J. Environ. Res. Public Health 18, 7573. 10.3390/ijerph18147573 34300024 PMC8306057

[B9] Faulhaber M. Pocecco E. Gatterer H. Niedermeier M. Huth M. Dunnwald T. (2016). Seven passive 1-h hypoxia exposures do not prevent AMS in susceptible individuals. Med. Sci. Sports Exerc 48, 2563–2570. 10.1249/MSS.0000000000001036 27414687

[B10] Forrer A. Scheiwiller P. M. Mademilov M. Lichtblau M. Sheraliev U. Marazhapov N. H. (2021). Exercise performance in central asian highlanders: a cross-sectional study. High. Alt. Med. Biol. 22, 386–394. 10.1089/ham.2020.0211 34432548

[B11] Fukuda T. Maegawa T. Matsumoto A. Komatsu Y. Nakajima T. Nagai R. (2010). Effects of acute hypoxia at moderate altitude on stroke volume and cardiac output during exercise. Int. Heart J. 51, 170–175. 10.1536/ihj.51.170 20558906

[B12] Gore C. J. Clark S. A. Saunders P. U. (2007). Nonhematological mechanisms of improved sea-level performance after hypoxic exposure. Med. Sci. Sports Exerc 39, 1600–1609. 10.1249/mss.0b013e3180de49d3 17805094

[B13] Hassan M. A. Weber C. Waitz M. Huang L. Hummler H. D. Mendler M. R. (2017). Reliability of pulse oximetry during progressive hypoxia, cardiopulmonary resuscitation, and recovery in a piglet model of neonatal hypoxic cardiac arrest. Neonatology 112, 40–46. 10.1159/000456648 28253519

[B14] Heinrich E. C. Djokic M. A. Gilbertson D. DeYoung P. N. Bosompra N. O. Wu L. (2019). Cognitive function and mood at high altitude following acclimatization and use of supplemental oxygen and adaptive servoventilation sleep treatments. PLoS One 14, e0217089. 10.1371/journal.pone.0217089 31188839 PMC6561544

[B15] Hetzler R. K. Stickley C. D. Kimura I. F. LaBotz M. Nichols A. W. Nakasone K. T. (2009). The effect of dynamic intermittent hypoxic conditioning on arterial oxygen saturation. Wilderness Environ. Med. 20, 26–32. 10.1580/08-WEME-OR-218.1 19364183

[B16] Holliss B. A. Burden R. J. Jones A. M. Pedlar C. R. (2014). Eight weeks of intermittent hypoxic training improves submaximal physiological variables in highly trained runners. J. Strength Cond. Res. 28, 2195–2203. 10.1519/JSC.0000000000000406 24513622

[B17] Inness M. W. H. Billaut F. Aughey R. J. (2017). Live-high train-low improves repeated time-trial and Yo-Yo IR2 performance in sub-elite team-sport athletes. J. Sci. Med. Sport 20, 190–195. 10.1016/j.jsams.2015.12.518 27142233

[B18] Jung M. Zou L. Yu J. J. Ryu S. Kong Z. Yang L. (2020). Does exercise have a protective effect on cognitive function under hypoxia? A systematic review with meta-analysis. J. Sport Health Sci. 9, 562–577. 10.1016/j.jshs.2020.04.004 32325144 PMC7749263

[B19] Kanz P. Krieger S. Drillich M. Iwersen M. (2018). Technical note: evaluation of a wireless pulse oximeter for measuring arterial oxygen saturation and pulse rate in newborn Holstein Friesian calves. J. Dairy Sci. 101, 6437–6442. 10.3168/jds.2017-14266 29705429

[B20] Katayama K. Sato Y. Morotome Y. Shima N. Ishida K. Mori S. (2001). Intermittent hypoxia increases ventilation and Sa(O2) during hypoxic exercise and hypoxic chemosensitivity. J. Appl. Physiol. 90, 1431–1440. 10.1152/jappl.2001.90.4.1431 11247944

[B21] Koehle M. Sheel W. Milsom W. McKenzie D. (2008). The effect of two different intermittent hypoxia protocols on ventilatory responses to hypoxia and carbon dioxide at rest. Adv. Exp. Med. Biol. 605, 218–223. 10.1007/978-0-387-73693-8_38 18085275

[B22] Komiyama T. Katayama K. Sudo M. Ishida K. Higaki Y. Ando S. (2017). Cognitive function during exercise under severe hypoxia. Sci. Rep. 7, 10000. 10.1038/s41598-017-10332-y 28855602 PMC5577198

[B23] Lefferts W. K. Babcock M. C. Tiss M. J. Ives S. J. White C. N. Brutsaert T. D. (2016). Effect of hypoxia on cerebrovascular and cognitive function during moderate intensity exercise. Physiol. Behav. 165, 108–118. 10.1016/j.physbeh.2016.07.003 27402021

[B24] Li J. W. O'Connor H. O'Dwyer N. Orr R. (2017). The effect of acute and chronic exercise on cognitive function and academic performance in adolescents: a systematic review. J. Sci. Med. Sport 20, 841–848. 10.1016/j.jsams.2016.11.025 28185806

[B25] Logan S. Tobin K. E. Fallon S. C. Deng K. S. McDonough A. B. Bavis R. W. (2016). Chronic intermittent hyperoxia alters the development of the hypoxic ventilatory response in neonatal rats. Respir. Physiol. Neurobiol. 220, 69–80. 10.1016/j.resp.2015.09.015 26444750 PMC4688092

[B26] Nagel M. J. Jarrard C. P. Lalande S. (2020). Effect of a single session of intermittent hypoxia on erythropoietin and oxygen-carrying capacity. Int. J. Environ. Res. Public Health 17, 7257. 10.3390/ijerph17197257 33020411 PMC7579477

[B27] Navarrete-Opazo A. Alcayaga J. Sepulveda O. Rojas E. Astudillo C. (2017). Repetitive intermittent hypoxia and locomotor training enhances walking function in incomplete spinal cord injury subjects: a randomized, triple-blind, placebo-controlled clinical trial. J. Neurotrauma 34, 1803–1812. 10.1089/neu.2016.4478 27329506

[B28] Netzer N. Strohl K. Faulhaber M. Gatterer H. Burtscher M. (2013). Hypoxia-related altitude illnesses. J. Travel Med. 20, 247–255. 10.1111/jtm.12017 23809076

[B29] Qiao Y. Liu Z. Yan X. Luo C. (2014). Effect of intermittent hypoxia on neuro-functional recovery post brain ischemia in mice. J. Mol. Neurosci. 55, 923–930. 10.1007/s12031-014-0447-8 25344154

[B30] Rieger M. G. Hoiland R. L. Tremblay J. C. Stembridge M. Bain A. R. Fluck D. (2017). One session of remote ischemic preconditioning does not improve vascular function in acute normobaric and chronic hypobaric hypoxia. Exp. Physiol. 102, 1143–1157. 10.1113/EP086441 28699679

[B31] Ritchie N. D. Baggott A. V. Andrew Todd W. T. (2012). Acetazolamide for the prevention of Acute Mountain sickness-A systematic review and meta-analysis. J. Travel Med. 19, 298–307. 10.1111/j.1708-8305.2012.00629.x 22943270

[B32] Rodriguez F. A. Casas H. Casas M. Pages T. Rama R. Ricart A. (1999). Intermittent hypobaric hypoxia stimulates erythropoiesis and improves aerobic capacity. Med. Sci. Sports Exerc 31, 264–268. 10.1097/00005768-199902000-00010 10063816

[B33] Rybnikova E. Samoilov M. (2015). Current insights into the molecular mechanisms of hypoxic pre- and postconditioning using hypobaric hypoxia. Front. Neurosci. 9, 388. 10.3389/fnins.2015.00388 26557049 PMC4615940

[B34] Shanjun Z. Shenwei X. Bin X. Huaijun T. Simin Z. Peng L. (2020). Individual chronic mountain sickness symptom is an early warning sign of cognitive impairment. Physiol. Behav. 214, 112748. 10.1016/j.physbeh.2019.112748 31770535

[B35] Shi Z. J. Cheng M. Liu Y. C. Fan X. R. Zhang Y. Wei Y. (2020). Effect of chronic intermittent hypobaric hypoxia on heart rate variability in conscious rats. Clin. Exp. Pharmacol. Physiol. 47, 60–66. 10.1111/1440-1681.13170 31454428

[B36] Shlim D. R. (2020). The use of acetazolamide for the prevention of high-altitude illness. J. Travel Med. 27, taz106. 10.1093/jtm/taz106 31897486

[B37] Tannheimer M. Lechner R. (2020). Rapid ascents of Mt Everest: normobaric hypoxic preacclimatization. J. Travel Med. 27, taaa099. 10.1093/jtm/taaa099 32577764

[B38] Taralov Z. Z. Terziyski K. V. Dimov P. K. Marinov B. I. Kostianev S. S. (2018). Assessment of the impact of 10-day intermittent hypoxia on the autonomic control measured by heart rate variability. Physiol. Int. 105, 386–396. 10.1556/2060.105.2018.4.31 30565474

[B39] Terada H. Hirata N. Sawashita Y. Ohno S. Yoshikawa Y. Yamakage M. (2021). Acute hypobaric and hypoxic preconditioning reduces myocardial ischemia-reperfusion injury in rats. Cardiol. Res. Pract. 2021, 6617374. 10.1155/2021/6617374 33815836 PMC7990552

[B40] Viscor G. Torrella J. R. Corral L. Ricart A. Javierre C. Pages T. (2018). Physiological and biological responses to short-term intermittent hypobaric hypoxia exposure: from sports and mountain medicine to new biomedical applications. Front. Physiol. 9, 814. 10.3389/fphys.2018.00814 30038574 PMC6046402

[B41] Wojan F. Stray-Gundersen S. Massoudian S. D. Lalande S. (2023). Brief exposure to intermittent hypoxia increases erythropoietin levels in older adults. J. Appl. Physiology 135, 88–93. 10.1152/japplphysiol.00172.2023 37262104

[B42] Woorons X. Richalet J. P. (2021). Modelling the relationships between arterial oxygen saturation, exercise intensity and the level of aerobic performance in acute hypoxia. Eur. J. Appl. Physiol. 121, 1993–2003. 10.1007/s00421-021-04667-8 33782716

[B43] Wu Q. Wu W.-S. Su L. Zheng X. Wu W.-Y. Santambrogio P. (2019). Mitochondrial ferritin is a hypoxia-inducible factor 1α-inducible gene that protects from hypoxia-induced cell death in brain. Antioxidants Redox Signal. 30, 198–212. 10.1089/ars.2017.7063 29402144

[B44] Wu Y. Zhou S. Li Y. Huang P. Zhong Z. Dong H. (2023). Remote ischemic preconditioning improves spatial memory and sleep of young males during acute high-altitude exposure. Travel Med. Infect. Dis. 53, 102576. 10.1016/j.tmaid.2023.102576 37068619

[B45] Yan Y. R. Zhang L. Lin Y. N. Sun X. W. Ding Y. J. Li N. (2021). Chronic intermittent hypoxia-induced mitochondrial dysfunction mediates endothelial injury via the TXNIP/NLRP3/IL-1β signaling pathway. Free Radic. Biol. Med. 165, 401–410. 10.1016/j.freeradbiomed.2021.01.053 33571641

[B46] Yang Q. Huang G. Tian Q. Liu W. Sun X. Li N. (2018). Living High-Training Low" improved weight loss and glucagon-like peptide-1 level in a 4-week weight loss program in adolescents with obesity: a pilot study. Med. Baltim. 97, e9943. 10.1097/MD.0000000000009943 PMC5842013 29465583

[B47] Yoon S. Kim B. R. Min S. H. Lee J. Bahk J. H. Seo J. H. (2021). Repeated intermittent hypoxic stimuli to operative lung reduce hypoxemia during subsequent one-lung ventilation for thoracoscopic surgery: a randomized controlled trial. PLoS One 16, e0249880. 10.1371/journal.pone.0249880 33857201 PMC8049270

[B48] Zhong Z. Zhou S. Xiang B. Wu Y. Xie J. Li P. (2021). Association of peripheral plasma neurotransmitters with cognitive performance in chronic high-altitude exposure. Neuroscience 463, 97–107. 10.1016/j.neuroscience.2021.01.031 33540052

